# A systematic review protocol of quantitative instruments of income inequality in studies of children and adolescents

**DOI:** 10.12688/hrbopenres.13456.2

**Published:** 2022-06-14

**Authors:** David O Driscoll, Elizabeth Kiely, Linda O Keeffe, Ali Khashan

**Affiliations:** 1 School of Public Health, Western Gateway Building,, University College Cork, Cork, Ireland; 2 School of Applied Social Studies, William Thompson House, Donovan's Road,, University College Cork, Cork, Ireland; 3 INFANT Research Centre, Cork University Hospital,, University College Cork, Cork, Ireland

**Keywords:** Income inequality, child health, poverty

## Abstract

**Background: **Income inequality is an important indicator of socioeconomic position which is a determinant of social, psychological, and physical health outcomes from childhood to adulthood. Different income inequality instruments (metrics) are used to investigate associations between income inequality and health outcomes (e.g. Gini coefficient). Income inequality instruments provide unique information on the construct of socioeconomic inequality. Albeit there is variation in studies as to the type and rationale for using a particular quantitative instrument of income inequality. The aim of this systematic review will be to investigate and identify the most used quantitative income inequality instrument in studies of children and adolescents up to 18 years of age.

**Methods: **The PRISMA-P framework will be applied to identify high quality articles (PROSPERO: CRD42021259114). A search will be conducted in PubMed, Embase, and PsycINFO. The search will include studies concerned with income inequality and/or socioeconomic inequality in children and adolescents. All articles will be independently reviewed, data extracted, and quality appraised by two

reviewers and a third to arbitrate disputes. Articles will be reviewed by title and abstract using inclusion criteria. A data extraction form will be used. Three questions will assess the quality of the rationale for using a particular income inequality instrument and the Newcastle-Ottawa Scale will be used to assess bias

and quality. The primary outcome of interest is the type and frequency of quantitative income inequality instruments used and the study outcome associated with that income inequality instrument.

**Conclusions: **This systematic review will aim to provide a summary of the different types of quantitative income inequality instruments used in studies of child and adolescent populations. This will help to guide researchers and policy makers on the use of income inequality metrics in future studies aimed at understanding associations with health and social outcomes in children and adolescents.

## Introduction and background

Income inequality demonstrates continual income gap between households that are either predisposed to or result in further social deprivation, evolving mental disorders, social injustice, poor education, poor employment attainment and lower life expectancy ( Buttrick *et al*., 2017; Chen *et al*., 2007; Coburn, 2000; Kawachi & Kennedy, 1997; Lillard *et al.*, 2015; Lynch *et al.*, 2000). Public and social policies (e.g. taxes, welfare benefits) may influence actual income level, perceived income level within a neighbourhood or effective income level on quality of life ( Kawachi & Kennedy, 1997; Kondo *et al.*, 2009; Morrissey *et al.*, 2020). Furthermore, addressing income inequality may benefit child and adolescent outcomes ( Engle *et al.*, 2011). Income inequality influences the mortality and health outcomes of children and their trajectories (longstanding illnesses, psychosocial wellbeing and obesity) into adolescence and adulthood ( Vallejo-Torres *et al.*, 2014). The effect of income inequality on child health outcomes differs throughout the life course, e.g. study findings differ as to the effect of income inequality on mortality based on age ( Lynch *et al.*, 2001; McIsaac & Wilkinson, 1997). Higher per person family income is associated with better outcomes in relation to physical activity, psychological symptoms and overall life satisfaction in adolescents. ( Elgar *et al.*, 2015). Moreover, there is evidence that those individuals in a higher income bracket are healthier and that addressing income inequality by raising the incomes of the poorest can improve health outcomes and health inequalities ( Lynch *et al.*, 2004)

Measuring inequality is a subject where a significant proportion of time is spent on conceptualizing inequality and the meaning of terms. The development of inequality within a region or the efficacy of a certain social reform can be documented differently depending on the instrument used to measure inequality ( De Maio, 2007). Therefore, due consideration must be given by researchers and policy-makers as to the metric of income inequality being utilised, in order to make accurate and well-informed associations with health outcomes. This includes the variables needed to calculate the income inequality instrument. Income inequality instruments (i.e. quantitative metrics of income inequality) include the Lorenz curve, the Gini coefficient, decile ratios, the Palma ratio, the Theil Index and others ( Trapeznikova, 2019). Each instrument provides insight into different aspects of income inequality. A policy-maker interested in the effect of a policy on the most socioeconomically deprived in a society may use the Palma ratio as an alternative to the Gini coefficient as their inequality instrument and concentrate on consumption instead of income data ( De Maio, 2007; Kawachi & Kennedy, 1999; Świgost, 2017). Moreover, inequality instruments across countries may vary, and differences exist in data sources and definitions (e.g. New-world bank classification of lower-middle, upper-middle, and high income) ( World Bank Country and Lending Groups, 2021). For example, measures of income inequality (e.g. data on income) are usually collected from household surveys and as such may not suit studying inequality at the top end of the income distribution as high-income respondents may be less likely to disclose all their wealth. In studies that focus on child outcome, each income inequality instrument provides additional information on the construct of socioeconomic inequality being investigated in relation to the measured child and adolescent outcome. The income data source used in child and adolescent studies is often at the household/family income level rather than individual level ( Choi *et al.*, 2017). Martikainen *et al.*, demonstrates that caution must be used in making associations between income types (e.g. household, individual, disposable) in mortality research and there is a need to better understand the role of individual income, household income and disposable income in understanding the association of income inequality and childhood outcomes ( Martikainen *et al.*, 2009). Moreover, income inequality instruments are not identical. A preliminary search of PubMed and Embase database did not yield any systematic reviews investigating the frequency of quantitative income inequality instruments used in outcome studies. Moreover, this illustrates that this review will be the first to report the frequency of use of each quantitative income inequality instrument in studies of children and adolescents.

The objectives of this systematic review protocol are to (a) determine the frequency of use of each quantitative (objective) income inequality instrument within studies investigating child and adolescent outcomes, (b) to ascertain if the frequency of quantitative income inequality instruments varies depending on characteristics (e.g. country, health outcome etc), (c) to determine the difference wealth sources (e.g. survey data, taxation data) used to calculate each income inequality instrument, (d) to discuss possible advantages and disadvantages of each income inequality instrument.

### Why perform this systematic review?

- The types of quantitative instrument of income inequality used in studies among children and adolescents is not well quantified.
- There is no consensus as to the most appropriate quantitative instrument of income inequality that should be used in studies among children and adolescents.
- The advantages and disadvantages of each method of defining and assessing quantitative income inequality instruments in studies of children and adolescents are not well understood.

## Methods

### Searches

**Figure 1.**
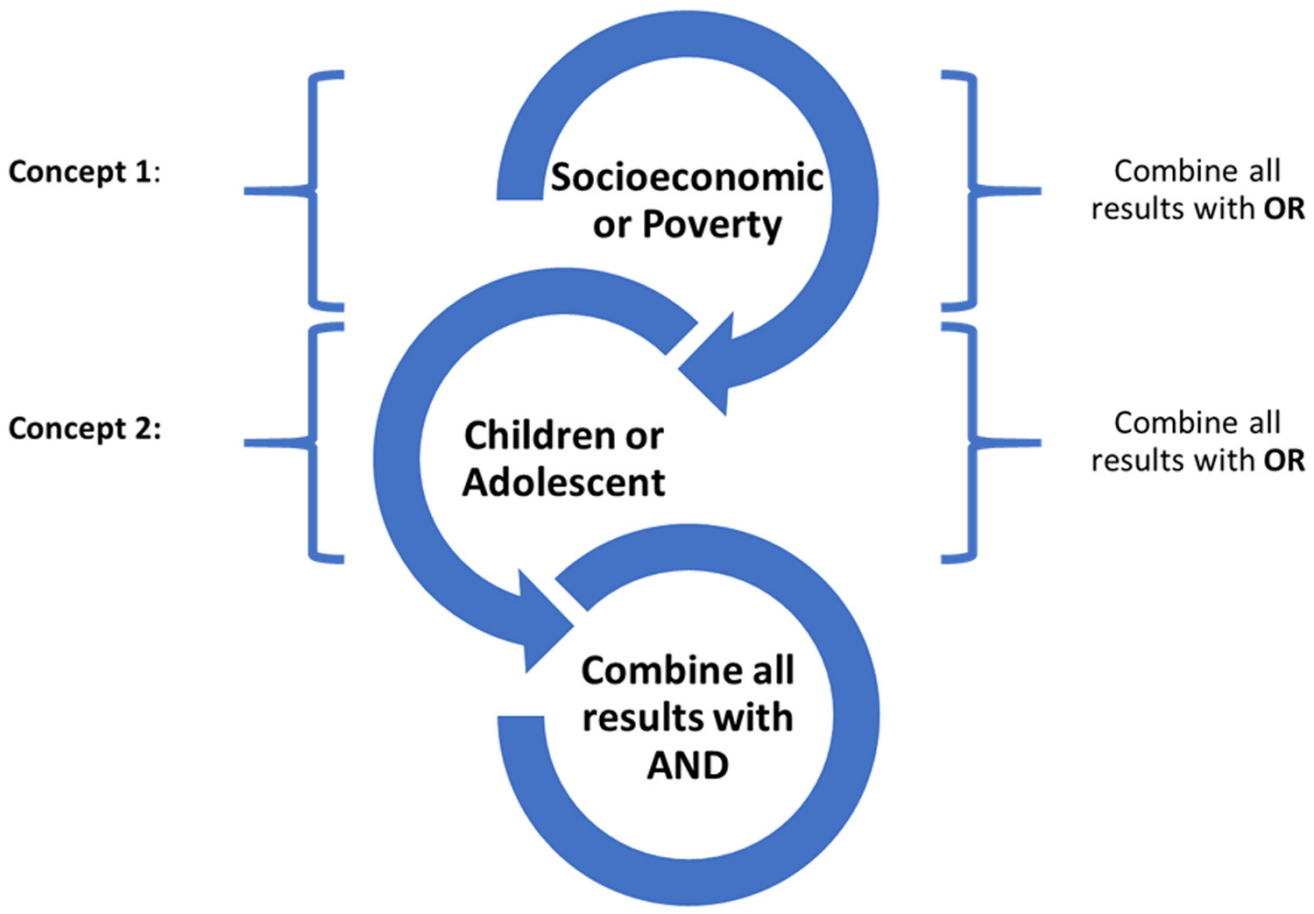
Search strategy.

The systematic review protocol is registered in the International Prospective Register of Systematic Reviews (PROSPERO) (ID: CRD42021259114 on 10/10/21). The following databases will be included in the search process: PubMed, Embase, and PsycINFO from 2010 up till January 2021. This is to capture countries of varying economic levels. The following key terms will be used in the search: “child/ren” or “adolescent/s” and “socioeconomic” or “poverty” or “social inequality” or “income”. ( Figure 1) A sample search strategy is available (see extended data).

### Types of study to be included

Inclusion criteria: quantitative study designs (cross-sectional, case-control, prospective, longitudinal, ecological) focusing on income inequality as the primary (main) study question in children and adolescents will be included (i.e. less than 18 years of age only). Articles published in the English language will be included and if published in any other language will be translated. Only published studies in peer-reviewed journals will be included.

Exclusion criteria: The following study designs (qualitative, case studies, randomised control trials, reviews, disucssions, ecological and commentaries) will be excluded as our interest is in the use of income measures in studies of child health outcomes. Grey literature and conference abstracts will be excluded. ( Table 1)

**Table 1.** Inclusion and exclusion criteria.

	Included	Excluded
1	Quantitative study designs in peer-reviewed journals (prospective, longitudinal, cohort, case control, cross-sectional, ecological).	Studies not published in peer- reviewed journals. Qualitative, case studies, randomized control trials, quasi experimental reviews, grey literature, conference abstracts, discussions, and commentaries.
2	Studies focusing on income inequality as the primary (main) study question.	Studies that only focus on income inequality as a secondary study question/outcome.
3	Child and adolescent population (<18 years of age).	Adult population (>18 years of age).
4	Articles in all languages.	Qualitative (subjective) measures of income inequality.

**Condition or domain being studied:**
  Instruments of quantitative income inequality, children and adolescents.
**Participants/population:**
  Participants will be children and adolescents (aged 0–18 years of age).
**Exposure(s):**
  Any exposure in a study that included the use of an instrument of quantitative income inequality.
**Comparator(s)/control:**
  No comparator.
**Main outcome (s):**
  Type of income inequality instrument used.
  Type of primary outcome (health outcome) associated with income inequality instrument.
**Additional outcome (s):** Not applicable.

### Data management

Selected articles will be stored and managed using Mendeley 1.19 Reference Manager Library. This will be used to facilitate sharing and collaboration between reviewers during the screening of abstracts and titles, data extraction and quality appraisal stages.

### Data extraction (selection and coding)

The following procedure will be used for data selection and data extraction. The titles and abstracts will be screened by at least two reviewers independently. A third reviewer will arbitrate if there are disagreements between the two reviewers. Abstracts that do not fulfil the inclusion criteria will be excluded. The abstract must contain and demonstrate that income inequality is a primary exposure of the study within the article. If there is uncertainty in terms of inclusion criteria, the article will be retained for the next stage of screening. Subsequently, full-text articles will be screened to ensure adherence to inclusion and exclusion criteria. This will be conducted by two independent reviewers. A third reviewer will arbitrate if there are disagreements between the two reviewers. Data extraction of selected studies will be done independently by two reviewers. Data extraction will include first author name, final author name, year of publication, journal name, origin of study location, study location (i.e. low, medium, high income), study setting (including if the instrument reflects national, regional or local level data), income inequality instrument(s), aim of study, sample population, age of population, sample size, frequency of income inequality measurement (e.g. one recording of salary or multiple recordings of salary), data collection method, analytical approach, statistical test, study design, primary outcome measured, and type of association reported ( Table 2). The primary outcome measured will be coded based on health outcome. A third reviewer will ensure accuracy of data extraction. References of included articles will be checked for any other potential eligible studies. The extracted data will be populated, categorised and stored in Microsoft Excel. A data abstraction form will be used ( Table 2). To improve the reliability of data abstraction by the reviewers, a pilot test will be performed of the data abstraction form on a small, random sample and if needed the form will be adjusted.

**Table 2.** Data Extraction Form.

First author name, final author name	
Publication type	
Year of publication	
Journal name	
Full reference	
Origin of study location	
Category of study location	
Study setting	
Income inequality instrument (1)	
Income inequality instrument (2)	
Aim of study	
Data source (e.g. survey)	
Sample population	
Age of population	
Sample size	
Frequency of income measurement	
Data collection method	
Analytical approach	
Statistical test	
Primary outcome measured	
Type of association reported	
Comments	

### Quality assessment

This review will apply the following critical appraisal tools based on study design (e.g. longitudinal) to assess the methodological quality of selected studies using the Newcastle-Ottawa Scale ( Stang, 2010). This systematic review will identify the frequency and type of income inequality instruments used. As such, the quality of the study is important, albeit the focus of quality assessment will be on whether a study explained the rationale for using a particular income inequality instrument. Moreover, three additional questions will be asked: (1) Does the study clearly state the income inequality instrument used? (2) Does the study explain the rationale for using that income inequality instrument? (3) Does the study explain how the income/consumption data was collected (e.g. survey, administrative data /taxation records) for that income inequality instrument? ( Table 3) This may facilitate further ability to categorise a studies explanation for using a particular income inequality instrument. Two reviewers will independently appraise the quality of the selected studies. Any discrepancies between reviewers will be discussed and resolved. A third reviewer will arbitrate if no consensus achieved. The consistency of the appraisal tool (Newcastle-Ottawa Scale) will be determined by calculating Cohen’s Kappa inter-rater reliability statistic ( Cohen, 1960). Studies will not be excluded based on quality of evidence, moreover it will be reflected in the narrative synthesis.

**Table 3.** Three questions to appraise the quality of rationale for using income inequality instrument.

**Did the study:**		
**1**	Clearly state the income inequality instrument used?	Yes ☐ No ☐
**2**	Explain the rationale for using that income inequality instrument?	Yes ☐ No ☐
**3**	Explain how the income/consumption data was collected (e.g. survey, tax)?	Yes ☐ No ☐

### Data synthesis

A qualitative meta-summary will be used to synthesise the descriptive findings from the quantitative studies. This is to apply a mixed research method synthesis that will aggregate and integrate the findings from the included studies.

### Ethics and dissemination

This research does not require ethical approval. It is retrospective in nature and does not involve direct or indirect research with human subjects. The research findings will be disseminated at conferences and published in an open access peer reviewed journal.

### Study status

At the time of publication of this protocol, database searches have been completed and study selection is underway. Completion of the review is expected by March 2022.

## Strengths and limitations

To the best of the authors’ knowledge, this review will be the first to systematically determine the prevalence of quantitative income inequality instruments in studies of the child and adolescent population. The methodological approach (e.g. extraction of data with a narrative synthesis) may provide a broader understanding to provide a comprehensive exploration of the topic. This exploration may highlight if researchers are using a particular income inequality instrument with specific health outcomes and if there is a rationale for same. The use of the Newcastle-Ottawa Scale in appraising the quality of the overall body of evidence will assist future readers in determining which quantitative income inequality instrument to utilise in their research when investigating outcomes in a child and adolescent population.

The authors anticipate that our research will have limitations. It is possible that relevant studies may not be found. The search time-period is limited and as such, it will not reflect the use of income inequality metrics prior to the inclusion dates. This research focuses on objective income inequality instruments, it does not include other important aspects of poverty including socioeconomic position (SEP), other environmental factors (e.g. neighbourhood poverty) or other scales (e.g. Family Affluence Scale). Grey literature, government reports and/or conference abstracts are not included. This review will focus on studies that include income inequality as a primary exposure. Income inequality measures investigated as a secondary exposure will not be included. This is to ensure the review is practical, achievable, and relevant.

## Conclusion

This protocol describes the methodological steps that will be taken in conducting a systematic review to identify and describe the quantitative instruments of income inequality. The thorough methodology to searching the literature, selecting studies, data extraction and appraisal, will better inform current and future research findings. The findings from this review will be valuable to stakeholders who are investigating or designing studies of income inequality or the effects of social inequality on child and adolescent outcomes. It may highlight additional varied instruments available to researchers and policy makers. Moreover, it may highlight the need for a cross-disciplinary discussion towards developing a standard conceptual framework for quantitative research on income inequality.

## Data availability

### Underlying data

 No data are associated with this article. 

### Extended data

Open Science Framework: “A systematic review protocol of quantitative instruments of income inequality in studies of children and adolescents **”**, https://doi.org/10.17605/OSF.IO/ABQ39 ( Driscoll (2021)).

This project contains the following extended data:

- Additional file 2 Preliminary Search Strategy.pdf

### Reporting guidelines

**Open Science Framework: PRISMA-P checklist for** “A systematic review protocol of quantitative instruments of income inequality in studies of children and adolescents”, https://doi.org/10.17605/OSF.IO/ABQ39 ( Driscoll (2021)).

Data are available under the terms of the Creative Commons Attribution 4.0 International license (CC-BY 4.0).
