# Supplementary material for: A systematic review protocol of quantitative instruments of income inequality in studies of children and adolescents
Source: HRB Open Res. 2022 Jun 14;4:132. Originally published 2021 Dec 24. [Version 2] doi: 10.12688/hrbopenres.13456.2 (PMC9327432; doi:10.12688/hrbopenres.13456.2)
Supplement: Peer review history

# Peer review: Reviewer response for version 2

**DOI:** 10.21956/hrbopenres.14821.r32318

**Published:** 2022-06-23

**Authors:** Emily Lowthian

**Affiliations:** 1 Population Data Science, Swansea University Medical School, Swansea, UK

Thank you to the authors for carefully responding to the peer reviewer comments I made. I am satisfied with the response, and I look forward to seeing the review indexed. Best of luck with the work.

Is the study design appropriate for the research question?

Yes

Is the rationale for, and objectives of, the study clearly described?

Yes

Are sufficient details of the methods provided to allow replication by others?

Yes

Are the datasets clearly presented in a useable and accessible format?

Not applicable

Reviewer Expertise:

Inequalities in child and adolescent health, adverse childhood experiences, parental behaviours, child wellbeing.

I confirm that I have read this submission and believe that I have an appropriate level of expertise to confirm that it is of an acceptable scientific standard.

# Peer review: Reviewer response for version 1

**DOI:** 10.21956/hrbopenres.14664.r31347

**Published:** 2022-02-10

**Authors:** Emily Lowthian

**Affiliations:** 1 Population Data Science, Swansea University Medical School, Swansea, UK

Overall, this protocol offers a promising and important question around income inequality measurement and child and adolescent outcomes. Research has shown that different measures of socioeconomic status often represent differing effects of inequality, e.g. parental education may be cultural, or knowledge-based, whereas income may represent material access. The protocol is clear to a large extent, but I think more can be done to really ground why focusing on child and adolescent income measurements are important - is the variation much of a concern when most studies have similar findings? The protocol seems to follow all of the correct guidance, but the guidance could be referred to more in the methods. Nevertheless, I look forward to seeing this systematic review published, and I imagine it will be important for research on children's inequality going forward. 

 I have some more detailed comments specific to each section that may improve the protocol:

**Introduction**

- The first paragraph is well structured, but could you give an example of how improving inequality may benefit outcomes? It may contextualise the work a bit better. 
- Could you find a reference, or give an example, of how different instruments are used across different countries?
- I think you can remove the brackets on "as high-income respondents..." - it would flow better, and the sentence well explains what you mean.
- The work would benefit from some examples of tools to measure child or adolescent inequality. In terms of child-reported measures, a common one used is the Family Affluence Scale. It is also worth discussing how income inequality may be differently collected among adults and children.
- The reasons to perform this systematic review are clear, but it might be worth honing in a bit more on how measures of income depict different aspects of inequality, e.g. total income may be more generalised compared to net income, which suggests more about the disposable income families may have.

**Methods**

- Minor, but could you be consistent in 'socio-economic' or 'socioeconomic' throughout the text and figures - this may also be important for your search strategy.
- On that, for your search strategy, you may want to include 'socioeconomic status', 'socio-economic status', 'SES', etc. to capture the full realm of how scholars inconsistently define it.
- More search terms for income inequality may be 'relative poverty', 'affluence', 'deprivation'. I appreciate you may want to slim down your search terms so this review is not huge!
- You may want to consider the term 'Young people' but screen out if they include people over 18?
- It may be worth citing your motives to exclude grey or third-sector literature as this could be very helpful, e.g. WHO definitions - but you are likely to pick this up in peer-reviewed journals too I hope. 
- Could you reference a definition of what low, medium, and high-income countries are so you are following an agreed understanding on this topic?
- Could you reference more standardised guidance in terms of the procedures you are conducting?
- I am assuming you will include mixed-method studies providing they have a quantitative element?

**Strengths and limitations**

- Could you draw on a systematic review that has researched socioeconomic status measures before, discuss the benefits of them, and then translate the intentions for your study?
- 'Limitations will exist' is a bit general. How about 'We anticipate that our research will have limitations in the areas of...''?
- I think the limitations section needs more work, e.g. studies that are not in English - that may exclude a large body of international knowledge, no grey literature, etc. They are fine limitations, but you need to be clearer in them.

Is the study design appropriate for the research question?

Yes

Is the rationale for, and objectives of, the study clearly described?

Yes

Are sufficient details of the methods provided to allow replication by others?

Yes

Are the datasets clearly presented in a useable and accessible format?

Not applicable

Reviewer Expertise:

Inequalities in child and adolescent health, adverse childhood experiences, parental behaviours, child wellbeing.

I confirm that I have read this submission and believe that I have an appropriate level of expertise to confirm that it is of an acceptable scientific standard, however I have significant reservations, as outlined above.

# Response

**Published:** 2022-05-26

**Authors:** David O Driscoll

**Affiliations:** University College Cork, Ireland

**Reviewer comment:**

 Overall, this protocol offers a promising and important question around income inequality measurement and child and adolescent outcomes. Research has shown that different measures of socioeconomic status often represent differing effects of inequality, e.g. parental education may be cultural, or knowledge-based, whereas income may represent material access. The protocol is clear to a large extent, but I think more can be done to really ground why focusing on child and adolescent income measurements are important - is the variation much of a concern when most studies have similar findings? The protocol seems to follow all of the correct guidance, but the guidance could be referred to more in the methods. Nevertheless, I look forward to seeing this systematic review published, and I imagine it will be important for research on children's inequality going forward. 

**Author response:**

 We thank the reviewer for their comments and for acknowledging that this piece of research may add to this research area.

**Reviewer comment:**

 The first paragraph is well structured, but could you give an example of how improving inequality may benefit outcomes? It may contextualise the work a bit better. 

**Author response:**

 Thank you for your feedback. We have added the following in the introduction: **“Moreover, there is evidence that those individuals in a higher income bracket are healthier and that by addressing income inequality will improve health outcomes and health inequalities (Lynch et al., 2004)”**

**Reviewer comment:**

 Could you find a reference, or give an example, of how different instruments are used across different countries?

**Author response:**

 We thank the reviewer for their insightful comment. We were unable to source a definitive reference to support that instruments may be different across countries. Albeit, the final systematic review paper results may be able to provide insightful evidence as to which instruments are being used in different studies within different countries.

**Reviewer comment:**

 I think you can remove the brackets on "as high-income respondents..." - it would flow better, and the sentence well explains what you mean.

**Author response:**

 We thank the reviewer for identifying this error. The brackets have been removed. 

**Reviewer comment:**

 The work would benefit from some examples of tools to measure child or adolescent inequality. In terms of child-reported measures, a common one used is the Family Affluence Scale. It is also worth discussing how income inequality may be differently collected among adults and children.

**Author response:**

 Thank you for your comment. We agree that additional measures are beneficial albeit this is not the focus of this systematic review. We have included an additional sentence to reflect that other measures do exist. We have included an additional line in main introduction text that certain studies would use household/family income when studying children rather than adults which can be household/individual income level. **“The income data source used in child and adolescent studies is often at the household/family income level rather than individual level (Young, 2017)”. **We intend to discuss this area in greater detail in the final systematic review paper.

**Reviewer comment:**

 The reasons to perform this systematic review are clear, but it might be worth honing in a bit more on how measures of income depict different aspects of inequality, e.g. total income may be more generalised compared to net income, which suggests more about the disposable income families may have.

**Author response:**

 We thank the reviewer for their insight on this area. We agree that there are different aspects of inequality based on income. We added the following to the introduction: **“Martikainen et al, demonstrates that caution must be used in making associations  between income types (e.g. household, individual, disposable) in mortality research and there is a need to better understand the role of individual income, household income and disposable income in understanding the association of income inequality and childhood outcomes (Martikainen, 2009).”**

**Reviewer comment:**

 Minor, but could you be consistent in ‘socio-economic’ or ‘socioeconomic’ throughout the text and figures – this may also be important for your search strategy.

**Author response:**

 Thank for identifying this inconsistency. We have changed all terms to socioeconomic in the protocol paper.

**Reviewer comment:**

 On that, for your search strategy, you may want to include ‘socioeconomic status’, ‘socio-economic status’, ‘SES’, etc. to capture the full realm of how scholars inconsistently define it.

**Author response:**

 We accept that there are inconsistent definitions. We are trying to focus our research piece on income only which is a part of socioeconomic status or position. As such, we will not be including socioeconomic status (SES) or socioeconomic position (SEP). We acknowledge this is a limitation and have included it in our limitations. Moreover, we are of the opinion this should be a separate distinct research project. **“This research focuses on objective income inequality instruments, it does not include other important aspects of poverty including socioeconomic position (SEP),  other environmental factors (e.g. neighbourhood poverty) or other scales (e.g. Family Affluence Scale)”**. Moreover, at present the topic is very broad with the current search strategy even without SEP/SES. As such, we are trying to make it practical in an area that is very broad. We will discuss this more in the final systematic review paper.

**Reviewer comment:**

 More search terms for income inequality may be 'relative poverty', 'affluence', 'deprivation'. I appreciate you may want to slim down your search terms so this review is not huge!

**Author response:**

 We agree with the reviewer. We acknowledge that there are many ‘relative’ terms used in the literature and for the purpose of this systematic review, we decided to limit it to objective metrics of income inequality. We will include this discussion and limitation in the final systematic review paper.

**Reviewer comment:**

 You may want to consider the term 'Young people' but screen out if they include people over 18?

**Author response:**

 We thank the reviewer for this insight. We agree that the term “Young People” is now more collectively acceptable. We did not include “Young people” as when optimising the search terms, it did not obtain additional search hits. 

**Reviewer comment:**

 It may be worth citing your motives to exclude grey or third-sector literature as this could be very helpful, e.g. WHO definitions - but you are likely to pick this up in peer-reviewed journals too I hope. 

**Author response:**

 Thank you for your comment. Yes, we excluded grey or third-sector literature. This was to ensure that the systematic review was practical and achievable. We agree with your comment that we most likely have obtained same through peer-review journals, which is the focus of this review. We acknowledge that it may be a limitation and have included the following to the limitations section: “ **We have not included grey literature, government reports and/or conference abstracts”.**

**Reviewer comment:**

 Could you reference a definition of what low, medium, and high-income countries are so you are following an agreed understanding on this topic?

**Author response:**

 Thank you for your comment, the definition we identified is based on the World Bank definition (2021-2022). https://blogs.worldbank.org/opendata/new-world-bank-country-classifications-income-level-2021-2022. The following sentence was added to the introduction for clarity: **“Moreover, inequality instruments across countries may vary, and differences exist in data sources and definitions (e.g. New-world bank classification of lower-middle, upper-middle, and high income) (World Bank Country and Lending Groups, 2021).”**

**Reviewer comment:**

 Could you reference more standardised guidance in terms of the procedures you are conducting?

**Author response:**

 Thank you for your comment. We highlighted the reporting guidelines (PRISMA-P checklist) under Reporting guidelines.

**Reviewer comment:**

 I am assuming you will include mixed-method studies providing they have a quantitative element?

**Author response:**

 Yes, mixed methods will be included provided they meet the inclusion criteria.

**Reviewer comment:**

 Could you draw on a systematic review that has researched socioeconomic status measures before, discuss the benefits of them, and then translate the intentions for your study?

**Author response:**

 We thank the reviewer for their comment. We did a preliminary search and did not identify a systematic review of quantitative income inequality instruments in our area. . The aim of this piece of work is limited and not including socioeconomic status or position measures. We agree, this is an important area and this is worth being part of a discussion in the final systematic review paper as a limitation.

**Reviewer comment:**

 'Limitations will exist' is a bit general. How about 'We anticipate that our research will have limitations in the areas of...''?

**Author response:**

 Thank you for your feedback. We have changed this sentence to **“The authors anticipate that our research will have limitations”.**

**Reviewer comment:**

 I think the limitations section needs more work, e.g. studies that are not in English - that may exclude a large body of international knowledge, no grey literature, etc. They are fine limitations, but you need to be clearer in them.

**Author response:**

 We have updated the limitations section to include additional limitations: **“The authors anticipate that our research will have limitations. It is possible that relevant studies may not be found. We have limited our search time-period and as such, it will not reflect the use of income inequality metrics prior to our inclusion dates. This research focuses on objective income inequality instruments, it does not include other important aspects of poverty including socioeconomic position (SEP),  other environmental factors (e.g. neighbourhood poverty) or other scales (e.g. Family Affluence Scale) . We have not included grey literature, government reports and/or conference abstracts.  This review will focus on studies that include income inequality as a primary exposure. Income inequality measures investigated as a secondary exposure will not be included. This is to ensure the review is practical, achievable, and relevant.”**

# Peer review: Reviewer response for version 1

**DOI:** 10.21956/hrbopenres.14664.r31105

**Published:** 2022-01-18

**Authors:** Richard M. Duffy

**Affiliations:** 1 Department of Psychiatry, Rotunda Hospital, Dublin, Ireland

O Driscoll *et al.,* have set out to undertake an important study on a topic that lacks clarity, yet is highly consequential. They have laid out their study protocol in this paper. Overall the methodology appears sound and the details of the study are clearly described.

 Small changes may improve the clarity of the protocol:

-  First aims b) and c) could be reworded to be clearer.
- On review of the search terms, there may be a role for expanding them, for example ‘minors’ and ‘wealth’ could be included.
- In the introduction or with the inclusion and exclusion criteria it would be interesting to see a brief discussion of subjective income inequality or the protocol could just state that it is only looking at objective income inequality. If it is looking at both this could be added to table 2.
- Table 2 could also include a reference to frame as income inequality can be measures at a national, regional or local level, this may be covered under the existing term ‘study setting’.
- In the inclusion criteria it would be helpful to be more explicit about how studies with minors and adults are dealt with. Also the authors could clarify how studies that have taken an ecological approach to look at income inequality are dealt with.

Despite these minor comments, this is a well written and clear protocol that is well designed.

Is the study design appropriate for the research question?

Yes

Is the rationale for, and objectives of, the study clearly described?

Yes

Are sufficient details of the methods provided to allow replication by others?

Yes

Are the datasets clearly presented in a useable and accessible format?

Not applicable

Reviewer Expertise:

The majority of my current work is on mental health law, but in the recent past I have researched subjective wellbeing variations during the recession, health outcomes in minority groups (asylum seekers and migrant populations).

I confirm that I have read this submission and believe that I have an appropriate level of expertise to confirm that it is of an acceptable scientific standard.

# Response

**Published:** 2022-05-26

**Authors:** David O Driscoll

**Affiliations:** University College Cork, Ireland

**Reviewer comment:**

 O Driscoll et al., have set out to undertake an important study on a topic that lacks clarity, yet is highly consequential. They have laid out their study protocol in this paper. Overall the methodology appears sound and the details of the study are clearly described.

**Author response:**

 We thank the reviewer for their feedback.

**Reviewer comment:**

 Small changes may improve the clarity of the protocol:

  First aims b) and c) could be reworded to be clearer.

**Author response:**

 We edited both (b) and (c) to provide greater clarity. **“…(b) to ascertain if the frequency of quantitative income inequality instruments varies depending on characteristics (e.g. country, health outcome etc), (c) to determine the different wealth sources (e.g. survey data, taxation data) used to calculate each income inequality instrument,**….”

**Reviewer comment:**

 On review of the search terms, there may be a role for expanding them, for example ‘minors’ and ‘wealth’ could be included.

**Author response:**

 We agree that the term wealth and minors may assist in the search scope, since we have begun the search, we will acknowledge that these terms were not included and may reflect a search limitation. This will be included as a limitation when the systematic review is completed.

**Reviewer comment:**

 In the introduction or with the inclusion and exclusion criteria it would be interesting to see a brief discussion of subjective income inequality or the protocol could just state that it is only looking at objective income inequality. If it is looking at both this could be added to table 2.

**Author response:**

 We agree that it is objective only. This has been made clear in the text. This has been included in the inclusion and exclusion criteria. **“….focusing on income inequality as the primary (main) study question in children and adolescents will be included (i.e. less than 18 years of age only).”**

**Reviewer comment:**

 Table 2 could also include a reference to frame as income inequality can be measures at a national, regional or local level, this may be covered under the existing term ‘study setting’.

**Author response:**

 We agree that this would be helpful and have included a comment under Data extraction in paracentesis **“…including if the instrument reflects national, regional or local level data”.**

**Reviewer comment:**

 In the inclusion criteria it would be helpful to be more explicit about how studies with minors and adults are dealt with. Also the authors could clarify how studies that have taken an ecological approach to look at income inequality are dealt with.

**Author response:**

 We have added an adjustment under Types of study to be included to make it more explicit the age profile **“…i.e. less than 18 years of age only”.** We have decided to exclude ecological approach studies as they do not use income data at individual level/household level which is the focus of this review  and added it to the exclusion criteria section **“Exclusion criteria: The following study designs (qualitative, case studies, randomised control trials, reviews, discussions, ecological and commentaries)”**

**Reviewer comment:**

 Despite these minor comments, this is a well written and clear protocol that is well designed.

**Author response:**

 We thank the reviewer for their insightful feedback and helpful suggestions to improve the quality of this piece of work.
